# Assessing Surgical Capacity in Guam: Current Strengths and Future Goals

**DOI:** 10.3390/ijerph23030353

**Published:** 2026-03-11

**Authors:** Ryan V. Benavente, Eduardo B. Biala, Brandon A. Lopez, Megan Y. Gimmen, Eric T. Pineda, John Reinier F. Narvaez, Russell K. Woo, Neal A. Palafox, Lee E. Buenconsejo-Lum

**Affiliations:** 1 Harvard Medical School, Harvard University, Boston, MA 02115, USA; mgimmen@hms.harvard.edu; 2 John A. Burns School of Medicine, University of Hawaii, Honolulu, HI 96813, USA; bialae@hawaii.edu; 3 University of Texas Medical Branch, The University of Texas System, Galveston, TX 77550, USA; braalope@utmb.edu; 4 Department of Internal Medicine, Elson. S. Floyd College of Medicine, Washington State University, Spokane, WA 99202, USA; epineda@tulane.edu; 5 Department of Surgery, Guam Regional Medical City, Dededo, GU 96913, USA; johnreinier.narvaez@grmc.gu; 6 Department of Surgery, John A. Burns School of Medicine, University of Hawaii, Honolulu, HI 96813, USA; russell.woo@hphmg.org; 7 Department of Family Medicine and Community Health, John A. Burns School of Medicine, University of Hawaii, Honolulu, HI 96813, USA; npalafox@hawaii.edu (N.A.P.); lbuencon@hawaii.edu (L.E.B.-L.)

**Keywords:** global surgery, health systems, equity, Micronesia, Guam, Pacific, Oceania, workforce capacity, surgical oncology

## Abstract

**Highlights:**

**Public health relevance—How does this work relate to a public health issue?**

- Guam serves as a regional area for surgical care in Micronesia; however, research regarding its surgical system is underrepresented.
- Information obtained from this study can be used to accurately assess Guam’s current surgical capacity, a necessary factor in the acute and chronic care of oncologic and non-communicable diseases, which has not been done before.

**Public health significance—Why is this work of significance to public health?**

- Identifies strengths and barriers in Guam’s surgical systems at two civilian hospitals and their ability to respond to the growing NCD/oncology burden and rising population from regional migration from across Micronesia.
- Without an understanding of the current surgical system and the factors necessary to improve it, the region is left vulnerable to natural disasters and mass casualty incidents, all of which may further strain the current surgical capacity in Guam.

**Public health implications—What are the key implications or messages for practitioners, policy makers, and/or researchers in public health?**

- There is an urgent need to invest in the infrastructure, workforce training, and specialty support to relieve the strain on Guam’s surgical systems to better expand equitable subspecialty surgical care for the native populations of the Pacific.
- Given its relative resources and development, there is an opportunity for Guam to respond as a surgical hub for the region of Micronesia, especially the oncologic burden and the rising threat of geopolitical conflict and natural disasters.

**Abstract:**

Introduction: Guam, the largest U.S. territory in Micronesia, plays a central role in surgical care for the local indigenous community and surrounding Pacific Island nations, yet remains underrepresented in surgical systems research. Methods: We conducted a mixed-methods study. Quantitative data were collected on operating volume, personnel, infrastructure, and surgical services at Guam Memorial Hospital (GMH) and Guam Regional Medical City (GRMC). Semi-structured interviews with hospital leadership and surgical providers captured qualitative insights on strengths, challenges, and future plans. Results: GMH and GRMC collectively provide general emergency, obstetric, and basic pediatric surgery, although advanced subspecialty and oncologic care remain limited. Although surgeons are highly adaptable with broad-practice capability, challenges, including resource limitations, aging facilities, advanced presentation, and subspecialty recruitment, limit the cases that are operable on Guam, resulting in expensive medical transfer. Anticipated stressors such as oncologic and non-communicable disease burden may further strain the system, emphasizing the necessity for modernized facilities and targeted recruitment of surgeons with regional ties. Conclusion: Strengthening Guam’s surgical capacity is essential for the provision of oncologic care and the advancement of health equity across the Pacific region, emphasizing an urgent need for investment in infrastructure, locally relevant workforce training, and regional policy development.

## 1. Introduction

Despite significant advancements in global health over the past 25 years, the world’s poorest regions continue to experience growing mortality and morbidity from conditions requiring surgical intervention [1,2]. Low-income and middle-income countries (LMICs) bear a disproportionately high burden of infectious disease, maternal disease, neonatal disease, non-communicable diseases, and injuries [2]. The diagnosis, prevention, and treatment of many of these diseases rely on the delivery of robust surgical and anesthesia care, which is integral to a functional and resilient health system in these communities. In response, the Lancet Commission on Global Surgery has established key benchmarks for LMICs to strive towards, including a surgeon, anesthesiologist, and obstetrician density target (SAO) of 20 per 100,000 and a surgical procedure rate of 5000 per 100,000 population, with the goal of improving essential preventive, diagnostic, and acute emergency care [2].

Micronesia is one of three ethnogeographic regions in the Oceania region. These islands face unique structural disadvantages, including their small and isolated populations, limited economies of scale, and shortages in the health workforce and infrastructure, which contribute to under-resourced health systems [3]. This region includes two U.S Flag Territories of Guam and the Commonwealth of the Northern Mariana Islands, as well as the three Freely Associated States of the Republic of Palau, the Republic of Marshall Islands, and the Federated States of Micronesia. Collectively, they are known as the U.S.-Affiliated Pacific Islands (USAPI) and represent a region with significant healthcare disparities [4]. Although updated data on surgical need in the Pacific is limited, Global Burden of Disease Data was used in 2015 to approximate that Oceania had 55,196 unmet surgical cases (555 per 100,000 persons), a number that has likely risen since then [5].

The surgical burden is predicted to further increase, particularly in the wake of prevalent non-communicable disease and rising cancer rates [1,4]. Lung, breast, oral, cervical, and prostate-related malignancies in Micronesia are common and are associated with several Micronesia-specific patient-specific social determinants of health, including poor diet, sedentary lifestyle, substance use, low cancer screening, medical mistrust, and pervasive socioeconomic barriers, resulting in high incidences of cancer at later stages of diagnosis [4]. Care delivery is hindered by persistent healthcare workforce deficits, exacerbated by an underfunded training infrastructure, limited resources, and geographic barriers that fragment care delivery and stretch limited clinical services across vast maritime regions [1,2,4]. Due to the inability to access timely elective care, surgical disease and sequelae worsen in complexity within these island settings, resulting in the need for off-island transfer or emergency evacuation, which often times is not financially feasible [1,2,3,4]. Consequently, mortality rates from cancer in these regions are far greater than the US average, particularly in Micronesian ethnic groups [4,6]. Furthermore, increasing natural disaster frequency secondary to climate change is expected to further fracture care and limit healthcare access, as infrastructure is destroyed and displacement increases, exacerbating the difficulties in providing care in Oceania [4].

Guam, the largest U.S. territory in Micronesia of approximately 167,000 people, comprises primarily Indigenous Pacific Islanders and Asian ethnic populations; Chamorros (32.8%) and Chuukese (6.7%) make up significant portions of the Indigenous Pacific Island population, while Filipinos represent the largest Asian subgroup (29.1%) [7]. Guam has two main civilian hospitals, Guam Memorial Hospital Authority (GMHA) and Guam Regional Medical City (GRMC), which care for the indigenous communities of Guam (50,420 persons), Compact of Free Association migrants (~18,000), and regional medical evacuations from the Micronesian region [4]. These communities are disproportionately subject to worse healthcare outcomes, resulting in greater rates of mortality, higher uninsured emergency room visits, and greater risks of experiencing delayed treatment [4].

Despite this responsibility, there is little documentation in the literature about the surgical capacity in Guam, which will need to confront this increase in volume and complexity. There have been other surgical assessments and scoping reviews on other Pacific island nations, but a formal assessment is needed for Guam, which remains the most populated island in Micronesia, a US territory that accepts regional migrants, a strategic military base, and an essential player in the surgical health of the region [1,4]. This assessment seeks to provide a comprehensive and respectful evaluation of both the existing strengths and the challenges to accessing surgical care in Guam.

## 2. Methods

Informed by priorities identified through preliminary consultation of local healthcare leadership, the authors conducted a needs assessment of the current surgical capacity at Guam’s two primary hospitals—Guam Memorial Hospital Authority (GMHA) and Guam Regional Medical City (GRMC), using a mixed methods approach. In accordance with the University of Hawai’i Office of Research Compliance’s Human Studies Program checklist, this study was determined to be exempt from IRB review as it did not meet the federal definition of human subject research. Permission to perform interviews and publish internal data was granted by the hospital leadership of GMHA and GRMC, with the plan to inform future quality improvement efforts.

A modified World Health Organization Surgical Assessment Tool was administered, completed, and returned by both institutions, with key capacity results being reported in Table 1. Based on the senior author’s prior policy and research work in the region, L.E.B-L. and N.A.P. suggested modifying the survey to facilitate completion by eliminating questions for which information was not readily available to the participants, the hospitals deemed as sensitive, or would not significantly add to the results gleaned from the other survey questions or thematic content analysis (Supplementary File S1). Using purposive sampling, from a pool of fourteen individuals, seven informants representing GMHA and GRMC, with an understanding of the clinical operations, infrastructure, and management of the civilian surgical system, were identified, including Chief Medical Officers (CMO), surgeons, and nursing staff. These interviews incorporated both structured and open-ended questions centered around access to timely essential surgery, specialist surgical workforce density, and surgical volume, core surgical indicators outlined by the Lancet Commission on Global Surgery, to supplement data on infrastructure and service delivery (Supplementary File S2) [2]. Due to the unavailability of data, perioperative mortality and financial metrics were not directly assessed. After 7 interviews, thematic saturation was reached, at which point no new themes were identified, and data collection was concluded.

Interviews were conducted using commercially available video teleconference platforms (Zoom version 6.4.6) and were recorded; transcripts were generated using said platforms’ cloud functionality. The interview script was not seen by the interview participant.

### 2.1. Data Analysis

Thematic analysis was utilized [8]. Individual transcripts were broken down into their constituent parts and open-coded by two team members (EB, MG). A third team member (RB) mediated any discrepancies. The individual codes were compiled into a standardized codebook. All personally identifiable information was removed from any code. Several iterations occurred during this process. As researchers with personal ties and interest in improving surgical care in the region, we acknowledge that our professional backgrounds may have influenced the framing of interview questions, the interpretation of participant responses, and the prioritization of certain themes over others. To mitigate the influence of these biases, themes were developed collaboratively across team members from different institutions outside of GMHA and GRMC, and disagreements in coding were resolved through discussion and consensus. Members from GMHA and GRMC were not involved in the coding of data. Furthermore, throughout the process, negative case analysis was performed to look specifically for contradicting data. These codes were eventually analyzed through an inductive process to identify four separate themes related to the current state of Guam’s surgical capacity; these were discussed by the authors of the paper at length and modified several times. Broad findings were then returned to participants to verify accuracy.

Descriptive statistics were used for demographics and outcomes. N (%) were used for categorical variables.

### 2.2. Data Management Methods

All data from the key informant interviews and the completed modified WHO survey were stored in a cloud-based spreadsheet on a secure drive with user-based login and password protection. No identifiable protected health information was collected.

## 3. Results

### 3.1. Characteristics of Guam Hospitals

Guam Memorial Hospital Authority (GMHA) is a public hospital in the central village of Tamuning with 161 beds, 4 operating rooms (OR), 6 post-anesthesia care unit (PACU) beds, 12 intensive care unit (ICU) beds, and 28 ventilators (Table 1). There are 3021 surgeries performed annually at GMHA. 39% of those surgeries are emergent. Five full-time surgeons, two full-time obstetricians-gynecologists (OB-GYN), and four anesthesiologists (plus six Certified Registered Nurse Anesthetists, CRNAs) handle the surgical case load. Notably, 225 of the annual cases are pediatric, despite the lack of access to a fellowship-trained pediatric surgeon. Health records are maintained through both electronic and paper mediums, and quality improvement projects, such as morbidity and mortality reviews, are performed on a monthly basis. Telemedicine is currently being implemented by GMHA’s ICU and Guam Behavioral Health’s local psychiatry services, connecting patients with off-island providers (Table 1).

Guam Regional Medical City (GRMC) is a private hospital in the northern village of Dededo with 150 beds, 10 ORs, 12 PACU beds, 14 ICU beds, and 12 ventilators. There are 2726 surgeries performed annually at GRMC. 58% of those surgeries are emergent. Ten full-time surgeons (including general, otolaryngology (ENT), orthopedic, urologic, and neurological specialties) and three anesthesiologists (plus three CRNAs) handle the surgical case load. Health records are maintained electronically, and general quality improvement projects are performed quarterly. Telemedicine is also currently being implemented to connect patients with off-island specialists (Table 1).

#### 3.1.1. Infrastructure

Anesthesia/Pharmacy: Both hospitals are equipped to provide inhaled, intravenous (IV), spinal, and regional anesthesia, with readily available supplies of paralytics, vasopressors, sedatives, and narcotics. GMHA notes some difficulties in maintaining adequate stores of IV fluids due to the national shortage.

Radiology: Computerized tomography (CT) imaging is available in both hospitals. Although available at GRMC, GMHA lacks the capacity to perform magnetic resonance imaging (MRI) studies. Scheduling ultrasound and X-ray procedures is sometimes challenging. Fluoroscopy will soon be available at GMHA. As of 2025, advanced endoscopy has become available to the island, where advanced procedures such as endoscopic retrograde cholangiopancreatography (ERCP) are now accessible and heavily utilized.

Blood supply and Laboratory: Both hospitals have the capacity to run basic electrolytes, coagulation profile, cardiac markers, complete blood count, crossmatch, and infectious panels, as well as provide blood transfusion within two hours.

#### 3.1.2. Service Delivery

Surgical Procedures: Surgeons at GMHA and GRMC perform all basic abdominal, urological, orthopedic, neurosurgical, and trauma procedures (Table 1). Both hospitals are unable to perform cataract extraction, trachoma treatment, or complicated cardiac and thoracic surgeries. Complex liver resections or procedures that require extensive use of platelets are limited to emergent cases due to logistical challenges that hinder platelet availability.

Obstetrics and Gynecology: GMHA provides obstetric and gynecological surgical care for Guam with full capacity for the provision of normal delivery, C-sections, emergency obstetric care, and cervical procedures (Table 1). GRMC does not offer any of these procedures, although there have been emergent gynecologic surgeries performed at GRMC because of ER patients being in extremis.

Pediatrics: GMH provides the majority of pediatric surgical procedures, with the capacity to treat a range of pathologies, including cleft lip/palate, anorectal malformations, and clubfoot. Only elective outpatient ENT, orthopedic, and urologic procedures are performed at GRMC, as there is no pediatric inpatient service to cater to more complex needs. As of 2022, GRMC has worked with Shriners Hospital for Children-Honolulu and the Guam Department of Public Health and Social Services to host short-term clinical services for pediatric orthopedic conditions, burns, and spinal cord injuries.

Theme 1: Strengths and Capabilities of Guam’s Surgical Workforce.

Participants cited multiple strengths that aid the workforce in being well-positioned to take care of the current Guam population. Surgical adaptability and resilience are among the most-regarded attributes among all participants. The surgeons of Guam have adopted a wide and varied practice to match the surgical volume of Guam. For instance, general surgeons in Guam are “true general surgeons” with competence in varied procedures from breast cancer resection to vascular to pediatric to oncologic surgery. This contrasts with the highly specialized role of the general surgeon in the continental US, but was born out of necessity. This willingness to care for the community has increased the reputation of the hospital systems in Guam, particularly during and after the COVID pandemic.

*“We are flexible. For example, even though I’m not a pediatric neurosurgeon, I do pediatric neurosurgery. We have general surgeons that are not pediatric general surgeons, but yet they do pediatric general surgery. So, I would say the strength is that we make ends meet somehow. If there is a need, we find a solution. And we work hard, and we are very innovative because we don’t have much. And so, the little that we have, we’re able to make things work.”*

In contrast to the scarcity of trained personnel experienced by both institutions only a few decades earlier, efforts to maintain a consistent staff of skilled general surgeons and surgeon subspecialists have greatly improved over time. In addition to a dedicated staff of “homegrown surgeons,” there is an increasing, active effort to recruit medical and surgical subspecialists with locum tenens privileges to fill capacity gaps. Furthermore, initiatives from nursing leadership directed at increasing local recruitment for ancillary staff have been largely successful, reducing the need for costly travel nursing services.

Additionally, there is a stable hospital infrastructure with reliable OR and inpatient facilities, X-Ray and CT imaging, and pathology services like frozen sections. Hospital leadership has also recently expanded its ability to track quality measures and has instituted a financial system that mostly protects its patients from impoverishment and catastrophic expenditures locally. Important elements within this system include outreach programs to enroll patients into Medicaid, protections for Compact of Free Association migrants to receive medical care, and a non-punitive financial culture.

All the aforementioned factors enable timely access to emergency procedures (~100% able to access emergent surgery >2 h), increased access to specialty elective care, including but not limited to surgeries longer than ten hours, and a larger surgical referral volume from the outpatient setting. Although access to patient mortality data was not granted, several interviewees cited comparable perioperative mortality standards to those in the continental U.S.

*“I don’t know what the regular rates are, but we rarely experience mortality in the OR. I mean, I think there’s only been a couple cases that I know of really in years. So, I want to say we are low risk.”*

Theme 2: Challenges in the Provision of Surgical Care in Guam.

Participants cited several difficulties in practicing in Guam. Longer delays in surgical presentation, resulting in more complicated pathology, comorbidity, and surgical planning, are common. Several participants attributed these observations to the underutilization of primary care, high care costs disincentivizing use of healthcare, limited medical literacy, and a local culture that avoids surgery and mistrusts the medical system. Cases are also more difficult due to rampant medical comorbidity, particularly non-communicable diseases like diabetes, hypertension, and heart disease.

The surgical and medical management of advanced surgical patients is complicated by a lack of specialty support and advanced ancillary staffing, areas of facility disrepair, and a tenuous blood product supply (blood is shipped and not collected within the hospital, and product storage for blood products like platelets and cryoprecipitate is limited). This results in certain patient groups, like burn or complex ICU patients, needing to be transferred elsewhere due to insufficient infrastructure.

*“Blood. That’s a problem. Massive Transfusion Protocol, for lack of a better term, it’s kind of a joke because we don’t have enough platelets. There was one instance I had a trauma patient who I did damage control surgery, needed MTP…We were lucky we had one platelet pack available, but we were only given half a pack because that other half was reserved for other medical patients that needed it. And that patient did okay, but say if the patient were to continue bleeding and required more MTP products, then we’d be screwed. So, the technical capability is there, but the resources.”*

Certain diagnostic services, like nuclear medicine, are minimal, while positron emission tomography (PET) is unavailable. Similarly, formalized pathology testing and processing for biopsy are shipped and performed off-island, resulting in a prolonged time to diagnosis.

Furthermore, surgical equipment and supplies are often limited or unavailable. Geographic isolation and suboptimal Pacific supply chains result in order delays, while recent tariffs and historical economic policies like the Jones Act and the Procurement Law are cited to drive up shipping costs and limit the flexibility of purchase. As a result, equipment and technology are often rationed or repurposed.

Several surgeons also cited low reimbursement rates from Medicaid as a financial difficulty for maintaining a practice in Guam. Furthermore, many local patients in Guam remain uninsured; high uninsured rates result in patients who either do not pay or declare bankruptcy, placing financial stress on patients, physicians, and the system alike.

Limited surgical subspecialist expertise, particularly for more complex oncologic and pediatric cases, and all cardiac cases, often results in greater off-island referrals, typically to the Philippines or the Western United States. These referrals are costly to families and the health system. In the event that this is not feasible, surgeons are often required to urgently operate beyond their scope of training or the available resources. Local general surgeons maintain a highly varied practice with a public expectation to be able to operate on cases typically assigned to surgical subspecialists (e.g., vascular surgery, pediatric surgery, colorectal surgery, and surgical oncology).

*“I would say technology is a major impediment to our work here in Guam. [In reference to neuromonitoring for neurosurgery] There is also an increased risk of the surgery. We explain to the patient, listen, we don’t have this, but you need to get this done. You can’t go off island because you have no passport. So, therefore, we’re going to do the surgery with you knowing that there is an increased risk of nerve injury. And then we’ll have to take more X-rays to make sure that the hardware is going in the right place. So good conversations is how we mitigate that.”*

Theme 3: Barriers to Recruitment for Surgeons and Specialists.

As cited by the majority of participants, there are not enough surgical specialists on the island of Guam to meet the demand and volume of the island. Factors that hinder recruitment include a lack of infrastructure to support specialized surgical programs, delayed adoption of prevailing surgical modalities such as robotics, a shortage of trained ancillary staff, inadequate equipment and facilities for particular procedures or post-operative pathways (e.g., cardiothoracic surgery or advanced oncologic surgery), as well as the high capital costs associated with initial establishment.

*“The main area where we are really missing, is cardiothoracic and I think that will, I think that will always be a challenge just because Guam really doesn’t have the infrastructure to support cardiac… maybe thoracic. A fully functional cardiac surgery team we you know, there would need to be a lot of investment into.”*

Student loan repayment programs are limited in Guam, despite its federal designation as a medically underserved rural area.

*“Loan repayment too. I mean, if not for my own personal situation, given my loans, it would be difficult for me to wholeheartedly go to Guam and practice my entire life. It’s an underserved area, but I looked into HRSA and National Health Service public health core repayment programs, and GRMC is not designated as one of those institutions that they would honor, which was really appalling.”*

While necessary, small specialty case numbers make it harder to justify the creation of these programs, especially from a technical skills maintenance and financing perspective. Furthermore, modern surgical training with a focus on specialization runs counter to Guam’s highly diverse and challenging surgical needs. Local surgeons have expressed concerns that newer graduates may not have the comprehensive training necessary to operate effectively in an environment like Guam. Finally, personal factors, including a small local applicant pool, lack of loan repayment, and geographic isolation, diminish Guam’s standing as a desirable place to work (Table 2).

Theme 4: Planning for the Future.

Participants cited multiple potential stressors to the hospital and health system, which are being evaluated. These include an increased case volume due to higher prevalence of late-stage disease, cancer, and population growth resulting from local military build-up, as well as concurrent civilian population increases. The anticipated increase in demand, concurrent with a decrease in surgical capacity due to aging surgeons without replacement and challenges in accessing supplies resulting from tariffs and dated trade policies, leaves the system vulnerable to being overwhelmed by massive systemic shocks, such as war and natural disasters.

*“The one big challenge that I could think is, you know, there’s currently there’s specific tensions. If it were to happen that there’s, you know, a war or a big disaster, I think it will, we will be ready, but it will definitely overwhelm not just our institution, but the island. So that’s my main worry.”*

Participants identified multiple future improvements of the system, including the procurement of new imaging modalities, achieving accreditation to treat more advanced or specialized pathology, investment in growing elective surgery, plans to procure new technology, the development of disaster response plans and allotment of disaster spending, discussions for GMHA to develop a new hospital with up-to-date facilities, and local partnerships to strengthen pre-hospital emergency systems and local academic research institutions (University of Guam).

*“I think the biggest one is we’ve got to invest in robotics as the next phase of investments for the island. And I think part of it, and the reason I say that it’s a must, because if you’re going to pulmonary, GYN, urology, general surgery, a lot of it is moving towards the robotics realm. And it’s a problem that if we don’t invest in it, we won’t have anybody who’s competent to do the surgeries.”*

**Figure 1:**
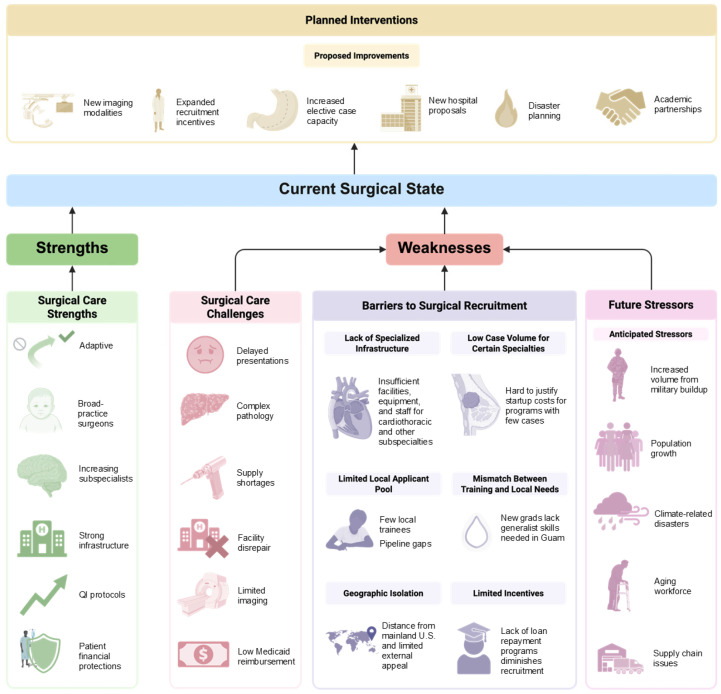
Current surgical state of Guam. Summary of the strengths and weaknesses of Guam’s current surgical capacity and infrastructure, based on thematically analyzed qualitative interviews from surgeons, nurses, and medical leadership within Guam Medical Hospital Authority and Guam Regional Medical City.

Aspirations also exist to increase inter-island-nation collaboration and referrals. Ways to actively recruit and increase retention of surgeons and ancillary staff have also been discussed, with improvements in reimbursement rates subsequently increasing the satisfaction of staff (Figure 1).

## 4. Discussion

### Surgical Capacity in the Pacific

Ricardo Eusebio, a well-known community surgeon in Guam, was among the first to highlight the challenges faced by rural, Pacific Island surgeons. The surgical system has come a long way since that seminal article [9]. Despite challenges such as geographic isolation, inconsistent access to medical resources and supplies, and a growing patient volume and disease burden, Guam’s surgical system remains remarkably adaptable and continues to improve. Our analysis demonstrates that Guam’s surgical system meets the Lancet Commission of Global Surgery’s benchmarks for timely access to emergent surgical care, stabilization, and treatment, as well as the minimum goal surgical workforce density of 20 providers per 100,000 (Table 3) [2]. The calculated total surgical volume of Guam does not meet the benchmark of 5000 procedures per 100,000, but is likely an underestimate, as this does not factor in elective outpatient surgeries and procedures performed at the private surgical center and the volume performed by the military hospital. Although quantitative data were not collected for perioperative mortality and catastrophic expenditure, efforts to improve both have been discussed in several interviews.

To the author’s knowledge, Palau is the only other Micronesian island that has had a formal capacity study conducted [1]. For a small community of approximately 18,000, Palau has a strong surgeon workforce (SAO density of 49.7), a surgical volume of 8606 per 100,000 (1557 surgeries annually), numbers that exceed Guam. However, there are reported resource limitations, including the inability to access blood, a lack of CT imaging, and some medication difficulties, which Guam meets in our preliminary analysis [1]. In addition, there is only one anesthesiologist physician in Palau, while pathologists and radiologists remain absent, representing a large gap in personnel that enhances diagnostic certainty [1].

In comparison to Fiji and Vanuatu, Melanesian islands more representative of Guam’s larger population, Guam demonstrates greater ability to meet surgical volume (Guam: 3441, Fiji: 2247, Vanuatu: 860), provide timely care (Guam: ~100%, Fiji: 67%, Vanuatu: 44%) as well as maintain SAO density (Guam: 21.6, Fiji: 7.1, Vanuatu: 3.2), although Fiji and Vanuatu have made great strides in improving their capacity overtime [1]. These findings demonstrate that regionally, Guam is performing well despite the challenges inherent to its geography, although it remains highly dependent on its territorial status.

Continued resilience of the medical system can partially be attributed to Guam’s talented and adaptable surgeons who have broad practice styles and increased recruitment of local ancillary staff for the pre and postoperative care of surgical patients. These characteristics, coupled with quality improvements in hospital protocols to meet more specialized accreditation standards, create a push towards offering more elective procedures, adopting advanced surgical technology such as imaging and robotics, and developing ambitious community partnerships with local health organizations. These are optimistic indicators for the island’s sustained growth.

*Workforce, Disease Burden, and Infrastructure Implications*

Difficulties with recruitment remain a significant concern, especially with anticipated increases in surgical volume over the coming decade [4,10,11]. Although the Guam SAO density of 21.6 narrowly meets the Lancet Global Commission of Surgery benchmark (>20 providers per 100,000), this can quickly change, particularly if providers retire or age out (Table 3). The population is increasing due to local military build-up and an influx of migrants from the FSM. Although the US Naval hospital exists to offset this influx, the entity largely cares for active-duty military, and dependents and contractors may start to rely on Guam’s civilian system. Furthermore, disease incidence is also expected to rise due to various cultural, socioeconomic, lifestyle, and dietary factors [4,10,11,12]. Non-communicable diseases are increasing at alarming rates throughout the Pacific, particularly diabetes and hypertension. Limited health literacy in combination with high costs often results in a reluctance to seek timely care [13,14]. Diseases often manifest in later stages of a pathology’s sequela, which result in more significant surgical intervention with a larger health burden on patients and the system, including amputation for peripheral artery disease, fistula creation for dialysis access for end-stage renal disease, and cardiovascular disease, which increases operative risk [4,15,16]. This is apparent in the hospital’s emergency surgery rates, which range from 39 to 58% of all surgical procedures, respectively (Table 1). To reduce comorbidity and the need for surgical intervention, continued public efforts to improve diet, exercise, health literacy, and primary care utilization and insurance enrollment, as well as the relationship between the health system and the historically marginalized indigenous populations of the Pacific, must be considered [4].

NCDs consequently contribute to the greater prevalence of cancer, which is reported to be increasing throughout Micronesia, especially in the Native Hawaiian and Pacific Islander population, resulting in the need for oncologic resection [4,15,16,17]. Betel nut and tobacco exposure in FSM, Guam, and Palau is associated with cancer, while radiation contamination from historical nuclear weapons testing has resulted in greater thyroid cancers in the Marshall Islands [4,18,19,20]. Presentation is often delayed due to fractured access to healthcare spread across dozens of islands, in addition to limited diagnostic screening and imaging, limited trained personnel, cultural barriers, and variable education on the value of surveillance [4,21,22,23]. All of these factors are anticipated to increase the oncologic case volume and case difficulty faced by Guam surgeons. With limited, incomplete, or delayed access to surgical care, patients are incentivized to fly off island, which is costly to the local medical systems and can be catastrophic to families. This can result in further delays in care and eventual mortality. A culturally informed public-health approach towards increasing the widespread utilization of early cancer screening modalities is recommended to reduce the burden of late-presenting disease in the future, in addition to outer-island outreach throughout Micronesia. Furthermore, there is a need for greater research on the unique environmental factors that specifically increase the risk of cancer in Micronesian populations, treatment failure, and the social, geographic, and economic barriers that prevent access to care.

*Regional Policy Implications and Future Directions*

In the context of escalating global conflict and the progression of climate change, massive system shocks such as mass casualty incidents and natural disasters can overwhelm the local infrastructure. In anticipation of these trauma events, thoughtful emergency planning and discussion will be helpful in the event of an acute surge in volume. While reliable medflight services exist to transport patients to the Philippines, Hawaii, Australia, or California, these transportation systems are limited in scope, as well as cost-prohibitive to families, as most local insurance carriers do not have medivac coverage [4,24]. The current literature reports that delays in surgical care result in greater mortality and morbidity, highlighting the need for more effective recruitment efforts in Guam, as it plays a central role in providing surgical care in the region of Micronesia [25,26,27].

As surgical care is currently strained throughout the Pacific, the Guam surgical system may have the future potential to serve as a more favorable alternative to seeking care outside of Oceania (in Asia and America), particularly as many Pacific Islanders have family throughout Micronesia, there is cultural understanding and support for unique customs, and the costs of flights are significantly less [1]. Local healthcare leaders and policymakers should explore multiple avenues to attract surgical talent to meet this increasing demand. These include sustained investment in surgical programs, encompassing infrastructure, technology, and ancillary staff, to attract specialists who can fill the elective surgical care gap and handle the anticipated increase in volume effectively (Figure 1). Lastly, and perhaps most importantly, physicians typically practice in locations where they trained or where they have personal or familial ties [28,29,30,31]. Although locum tenens surgeons have played an essential role in filling in gaps of care, this is a temporary solution. Regional policies that target recruitment should incentivize local surgeons and similarly positioned physicians to return and serve their communities, with the goal of creating a sustainable workforce pipeline.

## 5. Limitations

Our study has several limitations. The surveys were completed by a mix of healthcare leaders, including physicians and chief nursing staff, with many sections left incomplete. These include limited information on peri-operative mortality (although this is being measured) and patient expenditures (impoverishing vs. catastrophic). As a mixed-methods study, our findings are also limited by the perspectives of the surgeons and administrative staff we interviewed. Our focus was primarily centered on surgical leadership and their understanding of surgical needs, but including emergency physicians, primary care providers, and patients may have added greater depth to our study, particularly around questions centered on increasing patient complexity, financial hardship, and the perception of the system.

While the interviews followed a general framework, they were conducted by different team members, occasionally over multiple sessions. Follow-up questions varied and were not fully standardized, introducing potential interviewer bias. Additionally, the depth and openness of responses likely depended on the interviewers’ style and the participants’ willingness to engage. It is worth noting that, overall, participants were eager to speak openly about the current state of healthcare in Guam. Despite these limitations, there was a consistent alignment in perspective, with minimal disagreement regarding the current state of surgical care in Guam.

## 6. Conclusions

This study provides the first mixed-methods assessment of Guam’s surgical capacity, highlighting a resilient but resource-constrained system with strengths in workforce adaptability and emergency surgical care. Despite its unique challenges, Guam meets or approaches several benchmarks set by global surgical frameworks, positioning it as a potential surgical hub for the surrounding Pacific Islands. However, persistent challenges, including subspecialist shortages, infrastructure gaps, and difficulty recruiting and retaining surgical personnel, threaten long-term sustainability, especially as rates of resectable cancers increase. Addressing these issues will require investment in primary care and surgical infrastructure, locally tailored workforce training, and long-term policy solutions to recruit physicians with ties to the region. Strengthening Guam’s surgical system is not only essential for its population but for regional health equity in Micronesia and beyond.

## Figures and Tables

**Table 1:** Key services, volume, resources, staffing, and surgical characteristics of civilian hospitals on Guam.

Metric	Guam Memorial Hospital Authority (GMHA)	Guam Regional Medical City (GRMC)
Hospital Type	Public	Private
Location	Barrigada Heights	Dededo
Bed Capacity	161	150
Operating Rooms	4	10
Annual Surgeries	3021	2726
Emergent Surgeries (%)	39%	58%
Pediatric Surgeries	225	Minimal (circumcision only)
Capably Delivered OBGYN Services	C-section, vacuum extraction, aspiration and D&C, ectopic pregnancy care, tubal ligation, hysterectomy, cryotherapy for cervical lesions, and obstetric fistula.	No OBGYN services offered in the hospital
Capably Delivered Core General Surgery Services	Intestinal perforation, appendectomy, bowel obstruction, colostomy, gallbladder, hernia, hydrocelectomy, urinary obstruction, trauma resuscitation, airway, laparotomy, circumcision, and vasectomy.	Intestinal perforation, appendectomy, bowel obstruction, colostomy, gallbladder, hernia, hydrocelectomy, urinary obstruction, trauma resuscitation and laparotomy, circumcision, and vasectomy.
Capably Delivered Core Specialty Surgery Services	Amputations, skin grafting, burr hole, craniotomy, open reduction and internal fixation, irrigation and debridement of open fractures, tx for septic arthritis and osteomyelitis, club foot, cleft lip/palate, VP shunt, and anorectal malformation.	Amputations, skin grafting, burr holes, craniotomy, open reduction and internal fixation, irrigation and debridement of open fractures, tx for septic arthritis and osteomyelitis, and VP shunt.
Notable Surgeries Not Offered	Cataract extraction, trachoma	Cataract extraction, trachoma, pediatric surgery
Timely Access to Surgical Care Within Two Hours	~100%	~100%
SAO Density (part/full-time surgical, anesthetic, and obstetric physicians/100,000 population)	9.6 (16 physicians/per 167,000 persons)	12 (20 physicians per 167,000 persons)
Available Staff	Full-time surgeons: 5; full-time OBGYNs: 2; part-time OBGYNs: 5; anesthesiologists: 4; non-physicians performing anesthesia: 6; radiologists: 1; pathologists: 1; pharmacists: 17; biomedical technicians: 1.	Full-time surgeons: 10; part-time surgeons: 7; anesthesiologists: 3; non-physicians performing anesthesia: 3; pathologists: 1; pharmacists: 12; biomedical technicians: 5.
Electronic Records	Mixed (paper and electronic)	Fully electronic
Telemedicine Implementation	Ongoing	Ongoing
Quality Improvement Frequency	Monthly	Quarterly

**Table 2:** Barriers to surgical recruitment in Guam.

Barrier	Details
Lack of Specialized Infrastructure	Insufficient facilities, equipment, and staff for cardiothoracic surgery and other subspecialties.
Barriers to the Establishment of Certain Surgical Subspecialties	Large startup costs for specialty programs, including cardiac, transplant, and pediatric surgery, especially given the low—moderate volumes
Mismatch Between Training and Local Needs	New grads lack the generalist skills needed in Guam.
Limited Local Applicant Pool	Few local trainees; pipeline gaps.
Geographic Isolation	Distance from the continental U.S. and limited external appeal.
Limited Incentives (e.g., Loan Repayment)	Lack of loan repayment programs diminishes recruitment.

**Table 3:** Comparison of Guam Civilian Hospital’s surgical access, specialists, and volume to the Lancet Global Commission of Surgery 2030 landmark indicators.

Lancet Global Surgery Indicator	Global Benchmark	Guam Standard	Thematic Analysis of Key Stakeholder Perspectives	Representative Quotes
Access to Timely Surgery: Proportion of patients who can access a facility that can do a cesarean delivery, laparotomy, or stabilization of an open fracture.	80%	~100%	Strengths of Guam’s Surgical Workforce o Stable hospital infrastructure with reliable OR, provision of emergency services, and ancillary staffing (Table 1). o Maintenance of available, competent, general surgery staff. o Expansion of Medicaid enrollment and increased focus on patient financial protection. Challenges in the Provision of Surgical Care o Maintaining a stock of resources and repairing equipment. o Economic shocks to the supply chain. o Cultural mistrust of the medical system for patients, resulting in delay of care. o Limited diagnostic imaging modalities and pathology. Planning for the Future o Greater quality metrics tracking and improvements in continuing education. o Increasing elective case capacity.	*“Pretty much anyone who walks into the ER can access surgery in a timely maner.”* *“If it’s acute care surgery you’re talking about, there’s always a surgeon on call 24-. I would say it’s hard to put a percentage on it because technically it would be 100% if just by the fact that there’s a surgeon on the island at any given time, at least we can deliver surgical care that’s needed.”* *“100% except for cardiothoracic and urology and most pediatric emergencies. I would say 100% except for those three exceptions. Only because we don’t do urology here, and we don’t do cardiothoracic, and we don’t do complicated pediatric emergencies here. We can stabilize, but we won’t be able to do complicated pediatrics.”*
Specialist Surgical Workforce Density: Number of current, working surgeons, anesthesiologists, and obstetricians, per 100,000 population.	20	21.6 *	Strengths of Guam’s Surgical Workforce o Adaptive, broad practice surgeons. o Increasing recruitment of subspecialist physicians, including permanent staff and locum tenens. o Reduction in reliance on travel nursing, due to successful local ancillary staff programs. Challenges in the Provision of Surgical Care o Currently limited subspecialist expertise. o Lack of personnel. o Aging workforce. o Current gaps in the surgical recruitment pipeline (see Table 2). Planning for the Future o Expanded recruitment incentives to adjust for population growth.	*“I mean, the challenges of recruiting and retention, keeping them here in an old building, not having all the bells and whistles of a nice EHR, you know, or having equipment, you know, not being short on supplies and equipment. I think that’s a challenge because we know that our brothers and sisters that are working at [redacted for confidentiality] are working. Their working conditions are way better than ours. But I mean, a lot of us stay here because it’s about the people, right, and giving back to our community. So, for a lot of us, that weighs a lot more. So, the workforce is a challenge as well.”* *“Definitely a big need. The guys are getting older. We don’t really have any new guys; urology, Dr. [redacted], is the only guy right now. And he’s getting slammed. And so there is no urologist in Saipan or any other island. So, he’s pretty much the guy and all throughout Micronesia.”* *“I know of at least three general surgeons who are at the twilight of the career…And there’s a couple of them that have slowed down… you know, advanced age, so they need to be replaced. They’re not going to be here forever.”*
Surgical Volume: Procedures performed in the operating room annually, per 100,000 population.	5000 surgeries per 100,000 persons annually	3441.31 persons (5747 surgeries per 167,000)	Strengths of Guam’s Surgical Workforce o Improved reputation, from historical willingness to serve the community in times of high-volume (COVID). o Increasing recruitment efforts to meet increasing volume. o Maintenance of broad practice by general surgeons. Challenges in the Provision of Surgical Care o Rising rates of cancer and oncology rates. o Underutilization of primary care results in greater surgical complexity. o Reliance on off-island referral. Planning for the Future o Partnerships with EMS and strengthening of pre-hospital systems. o Disaster planning drills and contingency planning development in anticipation of climate change, MCI, and geopolitical conflict. o Development of inter-island and inter-nation partnerships.	*“There’s plenty of volume… I don’t have all the numbers off the top of my head…there’s definitely plenty of surgical volume.”* *“My understanding is that the Navy is planning to increase its capacity to meet the new Marines coming from Okinawa…head injuries are going to be a big issue. And if they don’t have a neurosurgeon, I don’t know how they’re going to meet that need. But right now, I would say that between GRMC and GMH and some of the outpatient clinics that are doing procedures, we are meeting the needs of the island for general surgery.”* *“Let’s start with the disease incidence because that seems to be the easiest to answer. Unfortunately, I think it’s going to get worse at the rate we’re going. So, I think as far as disease incidence, I think we’re going to see a lot more sickness. I don’t think that’s going to go away.”*

* Calculated from a population of 167,000 people.

## Data Availability

The original contributions presented in this study are included in the article/Supplementary Materials. Further inquiries can be directed to the corresponding author at rbenavente@hms.harvard.edu.

## Supplementary Materials

The following supporting information can be downloaded at: https://www.mdpi.com/article/10.3390/ijerph23030353/s1, File S1: Modified WHO Surgical Assessment Tool, File S2: Interview Guide.

## Abbreviations

The following abbreviations are used in this manuscript:

CMO	Chief Medical Officer
COFA	Compact of Free Association
CRNA	Certified Registered Nurse Anesthetist
CT	Computed Tomography
ENT	Ear, Nose, and Throat
ER	Emergency Room
ERCP	Endoscopic Retrograde Cholangiopancreatography
FSM	Federated States of Micronesia
GMHA	Guam Memorial Hospital Authority
GRMC	Guam Regional Medical City
ICU	Intensive Care Unit
IRB	Institutional Review Board
IV	Intravenous
LMICs	Low- and Middle-Income Countries
MRI	Magnetic Resonance Imaging
NCDs	Non-Communicable Diseases
OB-GYN	Obstetrics and Gynecology
OR	Operating Room
PACU	Post-Anesthesia Care Unit
PET	Positron Emission Tomography
USAPI	United States–Affiliated Pacific Islands
WHO	World Health Organization
