# Supplementary material for: Assessing Surgical Capacity in Guam: Current Strengths and Future Goals
Source: Int J Environ Res Public Health. 2026 Mar 11;23(3):353. doi: 10.3390/ijerph23030353 (PMC13026245; doi:10.3390/ijerph23030353)
Supplement: Supplementary file 1 [file ijerph-23-00353-s001.zip › ijerph-4162945-supplementary.pdf]

Supplementary Materials

Table of Contents

Modified WHO Surgical Assessment Tool -- 2-7

Interview Guide -- 8-9

Supplemental 1: Modified World Health Organization (WHO) Surgical Assessment Tool

The WHO surgical assessment tool has been repurposed and modified to look at key specific insights of Guam’s two civilian hospitals, GMH and GRMC. Modifications, primarily removals of questions, were made for the following reasons: 1) to facilitate ease of completion 2) Questions would otherwise be assessed in interviews and thematic analysis. 3) Questions are not relevant to the primary aims of the study 4) The data is not readily available and/or 5) the information may be sensitive to the hospital system. The following omissions are as follows:

- Questions pertaining to the maintenance of equipment were removed as these are assessed more qualitatively in the interviews and were removed for ease of completion of survey.
- Questions pertaining to the collection of data and research projects were removed as these were not our primary focus of our study and we asked more broadly in our interviews
- Questions pertaining to hospital financing, particularly budget and hospital out-of-patient costs were removed as these were not our primary focus of the study and the data was not readily available and was sensitive to the hospital system
- Questions pertaining to peri-operative mortality were removed as these were not our primary focus of the study and the data was not readily available and was sensitive to the hospital system

This document was delivered to both institutions and filled out by a trusted lead administrator This was subsequently returned. For privacy reasons, survey results are hidden but key findings are summarized in Table 1 and in the results section.

GENERAL QUESTIONS	
Country:	
Name of health care facility:	
Address of health care facility:	
Phone number of health care facility:	
Date of data collection (dd/mm/yyyy):	
Name and professional title of staff filling out form:	
Contact information of staff completing this assessment (email):	
Level of facility being evaluated	☐ Health Centre/Clinic ☐ District/Rural Hospital ☐ Provincial/Secondary Hospital ☐ Tertiary/Teaching Hospital
Type of facility being evaluated	☐ Public ☐ Private ☐ NGO ☐ Mission ☐ Other

Facility Characteristics	
Total number of admissions in a year	#
Total number of outpatients seen in a year	#
Total number of hospital beds	#
Total number of surgical beds	#
Total number of functioning operating rooms (major and minor)	#
Total number of post-anaesthesia care beds	#
Total number of advanced care/ICU beds	#
Total number of functional ventilators in the ICU	#
Access and referral systems:	
What is the population served by this facility?	#
What percentage of your patients can reach the hospital within 2 hours of travel?	☐ 0 ☐ 1-25% ☐ 26-50% ☐ 51-75% ☐ 76-99% ☐ 100%
What is the number of patients that you refer for surgical intervention to a higher level facility per year	#

INFRASTRUCTURE				
General Infrastructure - **How often is this item available <u>and</u> functional? Choose**<br>0 - Unavailable for Unavailable (**NOT AVAILABLE FOR ANYONE** who needs it);<br>1 - Inadequate (available to **LESS THAN HALF** of those who need it);<br>2 - Limited (available to **MORE THAN HALF**, but not to everyone who needs it); or<br>3 - Adequate (**PRESENT, AVAILABLE** to almost everyone in need, and used when needed).	Unavailable (0)	Inadequate (1)	Limited (2)	Adequate (3)
Dedicated 24 hour Emergency Unit	☐ 0	☐ 1	☐ 2	☐ 3
Electricity/operational power generator	☐ 0	☐ 1	☐ 2	☐ 3
Running water	☐ 0	☐ 1	☐ 2	☐ 3
Internet	☐ 0	☐ 1	☐ 2	☐ 3
Oxygen	☐ 0	☐ 1	☐ 2	☐ 3
Pharmacy-**How often is this available for surgery?**				
Inhalational general anaesthesia	☐ 0	☐ 1	☐ 2	☐ 3
IV sedation anaesthesia (Ketamine, Midazolam, Propofol)	☐ 0	☐ 1	☐ 2	☐ 3
Spinal anaesthesia	☐ 0	☐ 1	☐ 2	☐ 3
Regional anaesthesia available	☐ 0	☐ 1	☐ 2	☐ 3
Peri-operative antibiotics	☐ 0	☐ 1	☐ 2	☐ 3
IV fluids	☐ 0	☐ 1	☐ 2	☐ 3
Muscle relaxants/paralytics	☐ 0	☐ 1	☐ 2	☐ 3
Sedatives	☐ 0	☐ 1	☐ 2	☐ 3
Vasopressors	☐ 0	☐ 1	☐ 2	☐ 3
Post-operative narcotics	☐ 0	☐ 1	☐ 2	☐ 3

	Unavailable (0)	Inadequate (1)	Limited (2)	Adequate (3)
Radiology				
X-ray machine	☐ 0	☐ 1	☐ 2	☐ 3
Ultrasound	☐ 0	☐ 1	☐ 2	☐ 3
Fluoroscopy	☐ 0	☐ 1	☐ 2	☐ 3
CT scanner	☐ 0	☐ 1	☐ 2	☐ 3
MRI scanner	☐ 0	☐ 1	☐ 2	☐ 3
Blood Supply				
How often are you able to administer a blood transfusion within 2 hours	☐ 0	☐ 1	☐ 2	☐ 3
Laboratory				
Haemoglobin testing	☐ 0	☐ 1	☐ 2	☐ 3
Full blood count testing	☐ 0	☐ 1	☐ 2	☐ 3
Coagulation profile testing (PT, PTT, BT, INR)	☐ 0	☐ 1	☐ 2	☐ 3
Electrolytes testing	☐ 0	☐ 1	☐ 2	☐ 3
BUN and creatinine testing	☐ 0	☐ 1	☐ 2	☐ 3
Infectious panel testing (HIV, hepatitis virus, others)	☐ 0	☐ 1	☐ 2	☐ 3
Cardiac marker testing	☐ 0	☐ 1	☐ 2	☐ 3
Cross matching for blood and blood products	☐ 0	☐ 1	☐ 2	☐ 3

SERVICE DELIVERY

Rate adequacy as above.

0 - Unavailable for Unavailable (**NOT AVAILABLE FOR ANYONE** who needs it);
1 - Inadequate (available to **LESS THAN HALF** of those who need it);
2 - Limited (available to **MORE THAN HALF**, but not to everyone who needs it); or
3 - Adequate (**PRESENT, AVAILABLE** to almost everyone in need, and used when needed).

If less than adequate (rating 0, 1, or 2) then identify the barriers to access---→

Access Barriers
(Check all that apply)

Infrastructure - physical space, equipment or materials.
Absent - has never has been present
Broken –resources present, but broken
Personnel - resource, service or function available, and staff trained, but limited availability at times (eg, night, weekend or holiday)
Training - No staff trained in using resource or performing function
Stock out - cannot be procured, or required equipment or supplies out of stock often due to poor stock management practices or procurement failures
User fees - available, but out-of-pocket payment requirement prevents delivery for some
Other - Other factors

		Rate (0-3) 0-Unavailable 1-Inadequate 2-Limited 3-Adequate	**Infrastructure**	**Absent**	**Broken**	**Personnel**	**Training**	**Stock out**	**User fees**	**Other**
Procedures- Minor										
1. Normal delivery										
2. Suturing laceration										
3. Drainage of abscess										
4. Male circumcision										
5. Management of non-displaced fractures										
6. Wound debridement										
7. Removal of foreign body (throat/eye/ear/nose)										
8. Biopsy (lymph node, mass, other)										

Procedures – Major										
Obstetrics, gynaecology, family planning										
1. Caesarean birth										
2. Vacuum extraction/forceps delivery										
3. Ectopic pregnancy										
4. Manual vacuum aspiration and dilation and curettage										
5. Tubal ligation										
6. Vasectomy										
7. Hysterectomy for uterine rupture, intractable postpartum haemorrhage, or elective										
8. Inspection with acetic acid, cryotherapy for cervical lesions										
General Surgery										
9. Repair of intestinal perforations										
10. Appendectomy										
11. Bowel obstruction										
12. Colostomy/ileostomy										
13. Gallbladder disease										
14. Hernia, including incarceration										
15. Hydrocelectomy										
16. Relief of urinary obstruction: Catheterization or suprapubic cystostomy										
Injury										
17. Resuscitation with advanced life support measures, including surgical airway										
18. Tube thoracostomy										
19. Trauma laparotomy										
20. Open reduction and internal fixation										
21. Irrigation and debridement of open fractures										
22. Placement of external fixator										
23. Escharotomy/fasciotomy /contracture release										
24. Amputations										
25. Skin grafting										
26. Burr hole										
27. Craniotomy, not burr hole										
Non-trauma orthopaedic										
28. Drainage of septic arthritis										
29. Debridement of osteomyelitis										
Procedures - Advanced										
30. Repair obstetric fistula										
31. Repair of cleft lip and palate										

32. Repair of club foot										
33. Shunt/ETV/CPC for hydrocephalus										
34. Repair of anorectal malformation and Hirschsprung's Disease										
35. Cataract extraction and insertion of intraocular lens										
36. Eyelid surgery for trachoma										

Surgical Volume	
Number of laparotomies performed last year	#
Number of C-sections performed last year	#
Number of open fracture repairs performed last year	#
Total number of surgeries performed last year	#
Total number of paediatric surgeries (<15 years) performed last year	#
Percent of cases that were emergency/urgent (non-elective) cases	%

Quality and Safety	
WHO surgical safety checklist utilization in the operating rooms	☐ 0 ☐ 1 ☐ 2 ☐ 3
Pulse oximetry utilization in the operating rooms	☐ 0 ☐ 1 ☐ 2 ☐ 3
Hospital participation in quality improvement projects, such as mortality and morbidity conferences	☐ Never ☐ Monthly ☐ Quarterly ☐ Yearly

WORKFORCE

Surgeon/Anaesthesiologist/Obstetrician/Provider Density

**Providers**	**Full time**	**Part time**
Number of qualified surgeons	#	#
Number of qualified paediatric surgeons	#	#
Number of qualified OB/GYNs	#	#
Number of qualified anaesthesiologists	#	#
Number of general doctors providing surgery	#	#
Number of general doctors providing C-sections	#	#
Number of general doctors providing anaesthesia	#	#
Number of non-physicians providing surgery	#	#
Number of non-physicians providing C-sections	#	#
Number of non-physicians providing anaesthesia	#	#
Number of midwives	#	#
Number of nurses on the surgical wards	#	#
Number of qualified radiologists	#	#
Number of qualified pathologists	#	#
Number of qualified pharmacists	#	#
Number of qualified biomedical technicians	#	#

**Work Force Availability**	Unavailable (0)	Inadequate (1)	Limited (2)	Adequate (3)
Surgical provider availability	☐ 0	☐ 1	☐ 2	☐ 3
Obstetrical /gynaecology provider	☐ 0	☐ 1	☐ 1	☐ 3
Anaesthesia provider availability	☐ 0	☐ 1	☐ 1	☐ 3

Continuing Medical Education	
How often do you offer continuing medical education to your staff each year?	☐ Never ☐ Monthly ☐ Quarterly ☐ Yearly

FINANCING	
Health financing and accounting	
Percentage of patients with health insurance	□ 0 □ 1-25% □ 26-50% □ 51-75% □ 76-99% □ 100%

INFORMATION MANAGEMENT	
Information Systems	
What is the method of record keeping in your hospital?	□ Electronic □ Paper □ Both □ None
Are there personnel in charge of maintaining medical records?	□ Yes □ No
Are charts accessible across multiple visits for the same patient?	□ Yes □ No
Do you use telemedicine?	□ Yes □ No

Supplement 2: Interview Guide

The purpose of this interview guide is to systematically assess the current capacity, infrastructure, and challenges in maintaining the delivery of surgical care in Guam.These questions are meant to facilitate structured yet flexible discussions with key stakeholders to elicit descriptive, context-specific insights into daily operations, infrastructure, and community engagement that can inform future improvement efforts across the region. Interviews are not limited to these questions. Interviews will be 60 -90 minutes long.

Specific Aims/Objectives
This study aims to evaluate the surgical capacity of the non-military hospitals on Guam, which provide surgical care to the local population of the island, as well as the surrounding communities of Micronesia, and identify potential solutions and path forwards.

<u>Exploratory Questions Based on Six Surgical Indicators</u>

1. **Access to timely essential surgery**
    a. What percentage of your patient population are able to access essential surgery in a timely manner?
    b. What are the most significant barriers to accessing essential surgery
2. **Specialist surgical workforce density**
    a. What do you perceive are the biggest strengths of the surgical workforce in Guam?
    b. What do you perceive are the most significant gaps in the surgical workforce in Guam?
3. **Surgical volume**
    a. Can you describe how large the surgical volume is in Guam?
    b. Do you think there are enough surgeons to meet an influx of new residents in Guam? For instance, there have been plans to move the Marine base in Okinawa to Guam which would bring thousands of new residents to the island.
4. **Perioperative mortality (not really assessed in our survey)**
    a. How do you think perioperative mortality rates compare to mainland U.S.?
    b. What are some reasons you think is causing higher or lower rates?
5. **Protection against impoverishing expenditure (not really assessed in our survey)**
    a. What systems are in place to protect patients in Guam from impoverishing expenditures?
6. **Protection against catastrophic expenditure (not really assessed in our survey)**
    a. What systems are in place to protect patients in Guam from catastrophic expenditures?

<u>Exploratory Questions That Serve As Proxy Indicators For the Current Infrastructure</u>

1. Surgical Variety and Operational Capability
    a. Does Guam hospitals have the resources and staffing to handle a ten hour surgery?
    b. Questions Regarding Various Sub-Surgical Specialties:
        i. Can the hospital handle emergent pediatric problems?
        ii. Can the hospital handle the outcomes of hard surgeries?
        iii. Would you have a stent placed on Guam?
        iv. Would you do breast recon here?
        v. Would you get a total hip/knee here?
        vi. What are the barriers preventing specialists for coming here and/or from these programs to be developed?
    c. What forms of imaging are available? Which aren't?
    d. Can the ICU handle a difficult case/patient?

2. Systems and Utilities:
    a. Can we get live frozen? How quickly can pathology get back for graing for oncology cases?
    b. What is our imaging capacity?
    c. Is there always reliable access to electricity and water? Sanitation?
    d. Disaster response plans

Exploratory Questions That Investigate Adaptation to Challenges

1. What are the biggest issues that your hospital is currently facing and how have or will you plan to adapt to them?
2. How has this hospital and its surgical system changed in the past decade?
3. What do you anticipate being a challenge in the future, in regards to infrastructure, surgical capacity, or disease incidence? How are you planning to respond to this?
4. What are your future plans for this department of surgery?
5. What academic research or quality improvement initiatives are you most proud of?
